# The Effect of Bilateral, Two-Level Cervical Sympathetic Chain Blocks on Specific Symptom Clusters for Traumatic Brain Injury, Independent of Concomitant PTSD Symptoms

**DOI:** 10.3390/brainsci14121193

**Published:** 2024-11-27

**Authors:** Sean W. Mulvaney, James H. Lynch, Sanjay Mahadevan, Kyle J. Dineen, Kristine L. Rae Olmsted

**Affiliations:** 1 Department of Military and Emergency Medicine, Uniformed Services University, 4301 Jones Bridge Road, Bethesda, MD 20814, USA; james_lynch9@msn.com; 2 Orthobiologics Research Initiative Inc., 11200 Rockville Pike #230, North Bethesda, MD 20852, USA; sanjay@rosm.org (S.M.); kyle@rosm.org (K.J.D.); 3 RTI International, 3040 E Cornwallis Rd., Research Park, NC 27709, USA; krolmsted@rti.org

**Keywords:** stellate ganglion block, traumatic brain injury, neurobehavioral symptom inventory, posttraumatic stress disorder, two-level cervical sympathetic chain block

## Abstract

**Background/Objectives**: The aim of this study was to determine if performing ultrasound-guided, bilateral, two-level cervical sympathetic chain blocks (2LCSBs) (performed on subsequent days) improves symptoms associated with traumatic brain injury (TBI) that do not overlap with posttraumatic stress disorder (PTSD). **Methods**: A retrospective chart review was conducted between August 2022 and February 2023. We identified twenty patients who received bilateral 2LCSBs for PTSD and anxiety symptoms and who also had a history of TBI. Neurobehavioral Symptom Inventory (NSI) scores were collected at baseline, one week, and one month post treatment in 13 males and 7 females. A sub-analysis of the first ten questions of the NSI, which we identified as not overlapping with PTSD or anxiety symptoms, generated an NSI sub-score. **Results**: Out of 20 patients, all showed improvement in their NSI scores and NSI sub-scores. The NSI sub-scores had a baseline average of 15.45 (on a 40-point scale); the average score at one week post treatment was 8.30; and that at one month post treatment was 7.80. This represents a 49.51% improvement in TBI symptoms which did not overlap with PTSD or anxiety symptoms between baseline and one month. **Conclusions**: The use of bilateral 2LCSBs may be helpful in treating patients with TBI, regardless of the presence of comorbid PTSD symptoms.

## 1. Introduction

Traumatic brain injury (TBI) is a common pathology reported in both military and civilian populations, as 15–20 percent of returning service members report having sustained a mild TBI and up to 42 million people worldwide seek medical attention for mild TBIs every year [1,2]. TBIs may have varied clinical presentations among patient populations. The incidence of TBI in civilian populations is roughly 195 to 388 per 100,000 people and is more commonly seen in males [3]. There are approximately 27 million cases of TBI worldwide, and there is robust evidence to suggest that TBI may contribute to neurobehavioral sequelae [4,5]. Many in the scientific community consider concussions to be a form of mild TBI, and there are studies to suggest that the incidence of concussions is underreported in some populations [6,7]. In military communities, some estimates suggest that upwards of 28 percent of service members experience TBI [8]. In a study of nearly 2 million U.S. veterans, Stewart and colleagues reported that even a moderate TBI was related to the development of brain cancer [9]. Additionally, there is evidence to suggest that military-related TBI raises the likelihood of suicide, posttraumatic stress disorder (PTSD), or worsening of mental health conditions [10]. Studies show that soldiers returning from combat deployment with previous TBI had a 43.9% incidence of concurrent PTSD [11].

TBI may arise from multiple categories of injury. Closed-head TBI’s are the most common category of TBI from blunt force traumas (such as a fall or athletic impacts) [12]. The blunt force incident may invoke either rotational or linear stretch of neurological tissue, which damages the axon cords of cells and may disrupt cerebral blood flow [13]. More specifically, studies suggest that microtubules are the weak link of the axonal cytoskeleton and most likely to be damaged during traumatic axonal injury due to the rapid stretching of these structures that occurs during a concussion [14,15]. Furthermore, microtubule damage as sequelae of TBI or concussions is suspected to cause tau protein accumulation through prion-like spread and is associated with other neurodegenerative conditions like chronic traumatic encephalopathy and Alzheimer’s disease [16,17,18,19]. Penetrative TBIs result from a projectile entering through the skull and through neurologic tissue, which is sheared and ruptured [20]. Penetrating TBIs are often considered highly concerning events due to potential catastrophic failure of essential organ systems in an afflicted patient [20]. As previously discussed, much of the TBI-related research is associated with military populations during and following the Global War on Terror [21]. Many soldiers deployed to combat report experiencing blast-related TBI from exposure to ordinance or other ballistic weapon use [22]. Blast-related TBIs result from the sudden movement of pressure shock through the skull and brain tissue, with the kinetic energy of the blast damaging cellular structures [23,24]. These injuries may include components of penetrative vs. closed TBI depending on secondary materials, such as foreign projectiles, which may move towards a patient. While the category of TBI may differ, breakdown of brain barriers, neuronal injury, mitochondrial dysfunction, and persistent neuroinflammation all contribute to post-injury symptoms [25,26,27]. The symptoms of TBI may have rapid or prolonged onset, which may be indicative of severity, and symptoms may persist for months or years without resolution [28,29,30].

We have previously described the interlinked relationship between TBI and PTSD [31]. Notably, both conditions share some overlapping symptoms, etiology, and pathophysiology. Physical events or impacts may generate both TBI and PTSD in patients, although PTSD may also have eventual onset due to psychosocial factors that act as a stimulus [32]. Both PTSD and TBI are known to change brain activity and function by altering neuroinflammatory, oxidative, and excitotoxic mechanisms via disruptions to neural circuitry [33]. These tissue-level disruptions are thought to result in abnormalities of the fronto-cingulo-parietal cognitive control network, manifesting in behavioral and neurological changes in the typical function of cognition, memory, attention, and inhibition of fear processing [34]. Unfortunately, conservative care often falls short and fails to address the underlying mechanisms of both conditions with a tendency toward persistence and chronic degenerative cognitive changes over time [35].

Due to the large patient population affected by TBI, there is a need to develop novel therapeutic and interventional treatment options for patients. The stellate ganglion block (SGB) is a procedure that has been used since the 1920s to treat a variety of conditions, primarily pain-related. In 1990, Lebovits et al. [36] first described the effects of stellate ganglion blocks on improving symptoms of PTSD. Since that time, there have been over thirty-five peer-reviewed publications demonstrating its safety, utility, and patient acceptability in the treatment of PTSD [37]. The stellate ganglion is a part of the cervical sympathetic chain, which is located along both sides of the neck. The stellate ganglion is a cluster of nerves located in proximity to the C7 vertebra and is a result of the fusion of the inferior cervical and first thoracic sympathetic ganglia. It is thought to play a significant role in mediation of the sympathetic nervous system [38].

There is significant anatomic variation in the cervical sympathetic chain that may result in an incomplete block of the cervical sympathetic chain with a single injection at the sixth cervical vertebrae level only [39,40]. To address this fact, in our 2020 study, a novel ultrasound-guided two-level cervical sympathetic chain block was performed at the levels of the fourth and sixth cervical vertebrae and demonstrated superior improvements in PTSD symptoms compared with a single-level SGB at the sixth cervical vertebra [41]. These findings were repeated in a later study in 2022 by Lipov et al. [42]. Although SGB remains the commonly accepted term for this procedure, “cervical sympathetic block” better describes this procedure. We prefer to use a two-level cervical sympathetic chain block (2LCSB) as a more accurate term since this procedure targets the sympathetic chain and does not specifically target the stellate ganglion.

Initially, the first two case series documenting the use of SGB to treat PTSD described the procedure being performed on the right side at the level of the sixth cervical vertebra [43,44]. Our selection of exclusive treatment of the right side was based on the understanding of the crucial role that the right cerebral hemisphere plays as a part of the central and autonomic nervous system stress response [45,46]. Per our 2015 Clinical Practice Guidelines publication, we began performing left-sided SGB only by exception if the right-sided SGB failed to result in clinical benefit in PTSD [47]. In 2019, Rae Olmsted et al. [48] published a multi-center randomized controlled trial (RCT) demonstrating the effectiveness of right-sided SGB procedures spaced two weeks apart for the treatment of PTSD symptoms. As such, the SGB procedure was deemed a potential adjunct to trauma-focused psychotherapy and has been readily adopted in many treatment centers over the past 10 years [49].

In 2021, we published the first case series demonstrating that 5 percent of patients with PTSD only responded to a left-sided SGB in comparison to right-sided SGB. Up until that point, only the right side had been reported in the medical literature for the treatment of PTSD [50]. In response to this finding, we began routinely incorporating bilateral 2LCSB for the treatment of PTSD. Right- and then left-sided 2LCSBs were performed on subsequent days to eliminate the risk of inadvertent bilateral recurrent laryngeal nerve block and subsequent potential respiratory distress. Our 2023 study, the first report of durable improvements in anxiety symptoms, as measured by improvements in Generalized Anxiety Disorder 7-item Scale (GAD-7) [51] scores, showed that bilateral 2LCSBs are both safe (when each side is performed on subsequent days) and can result in greater improvements in GAD-7 scores than in a right-sided procedure alone [52].

Upon incorporating bilateral 2LCSBs, we noted that in addition to the expected improvements in PTSD symptoms during routine follow-up, both headaches and brain fog were clinically improved in our patients with a history of TBI. This observation prompted us to include the Neurobehavioral Symptom Inventory (NSI) [53] in patients with a previous diagnosis of chronic TBI, in addition to our usual screening of follow-up with the Posttraumatic Stress Disorder Checklist for DSM-5 (PCL-5) [54] and GAD-7 [55]. The NSI has value for clinicians and researchers as the gold standard in characterizing the presence and severity of symptoms and tracking symptomatic change in individuals with TBI, even though it is not directly intended to diagnose TBI [56,57]. The NSI has been adopted by the U.S. Department of Defense and the Department of Veterans Affairs for both research and clinical evaluation of TBI [57].

The underlying mechanisms for 2LCSBs improving TBI symptoms are unknown. However, there is initial evidence suggesting SGB works to improve TBI-related symptoms through modulation of brain inflammatory factors like NF-kB, regulation of nerve–endocrine–immune system dysfunction, and increase in perfusion patterns in the cerebral hemisphere to improve dysfunctional perfusion [58,59,60].

## 2. Materials and Methods

This retrospective case series was approved by the institutional review board of the Institute of Regenerative and Cellular Medicine (IRCM-2023-358). The twenty patients in this retrospective case series underwent 2LCSB treatment for PTSD symptoms, not for treatment of TBI symptoms. These patients all had a prior history and diagnosis of PTSD. Our inclusion criteria for PTSD diagnosis included a behavioral health provider’s formal diagnosis and a score of 40 or greater on the PCL-5 [54]. The PCL-5 is considered the standard for self-reported assessment of PTSD symptoms and has strong support in terms of psychometric application [61,62,63,64].

Twenty patients with prior history of PTSD and mild to moderate TBI diagnosis before treatment underwent bilateral 2LCSB treatment between August 2022 and February 2023. All patients completed patient-reported outcome measures in the form of the PCL-5, the GAD-7, and the NSI at baseline, one week, and one month post 2LCSB. The 20 items on the PCL-5 are scored on a scale of 0 to 4, with a range of 0 to 80. Likewise, the NSI is scored on a scale of 0 to 4, with 22 items and a range of 0 to 88. The GAD-7 has 7 items, each scored on a scale of 0 to 3, with a range of 0 to 21. The PCL-5 and NSI were provided to patients either through a secure email system or via paper copy; baseline, one week, and one month PCL-5, GAD-7, and NSI scores were manually entered into a secure data collection platform.

The 2LCSB procedure was conducted in adherence to the strict procedural guidelines associated with prior published works. This strict protocol adherence ensures that all patients receive an effective block and that the findings are reproducible across past and future studies. The following text describes the procedure in full detail:

Per published guidelines, all patients had a bilateral 2LCSB at the 4th cervical vertebra and 6th cervical vertebra, with the right side performed on day 1, and the left side performed on day 2 [50]. The blocks were performed on subsequent days to eliminate the risk of inadvertent bilateral blockade of the recurrent laryngeal nerve and subsequent potential airway compromise. A Doppler ultrasound scan was utilized prior to every procedure to clearly identify the vertebral artery and vein, as well as other vasculature. A 50-mm 25-gauge needle was utilized under ultrasound-guidance (General Electric Logic e with an 8–12 MHz broadband linear transducer) using a lateral, in-plane approach at both the 6th cervical vertebra level (using 6–8 mL of 0.5% ropivacaine) and the 4th cervical vertebra level (using 1.5–2 mL of 0.5% ropivacaine). All procedures were performed at an established musculoskeletal practice by pain and sports medicine fellowship-trained physicians who have collectively performed more than 5000 SGBs. Horner’s syndrome, a sign of a successful blockade of the stellate ganglion, is characterized by ptosis, miosis, and scleral injection and was scored by two independent observers at 5 min post-block per published guidelines [47]. All patients met the minimum clinical threshold for an acceptable Horner’s syndrome of 4 out of 6 points by both observers.

We conducted a retrospective sub-analysis of the first ten questions of the NSI that we identified as TBI symptoms which did not (in most, but not all cases) overlap with PTSD symptoms. A composite score of these non-overlapping questions was generated and denoted as an NSI sub-score, with the maximum possible score being 40. The non-overlapping questions identified as specific to TBI included prior symptomatic history of dizziness, loss of balance and coordination, headaches, nausea, vision problems, hearing difficulty, sensitivity to light and noise, and numbness or tingling of the body.

## 3. Results

All twenty patients treated with bilateral 2LCSBs in our retrospective analysis saw improvement in NSI scores. The average NSI baseline was 44.50 (41.54 for males and 50.0 for females), which decreased to 20.00 after one week (16.38 for males and 26.71 for females), and had an outcome of 22.40 after one month (18.85 for males and 29.0 for females). The total NSI scores across these 20 patients revealed similar decreases in raw score and percent improvement from baseline.

**Figure 1:**
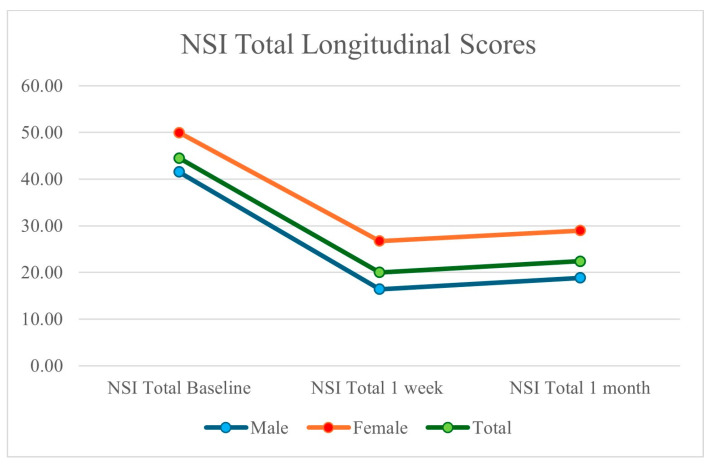
Decrease in patient NSI scores following 2LCSB intervention at baseline, one week, and one month. Total scores decreased by nearly 50%, while male patients improved by nearly 10% more than female patients.

**Figure 2:**
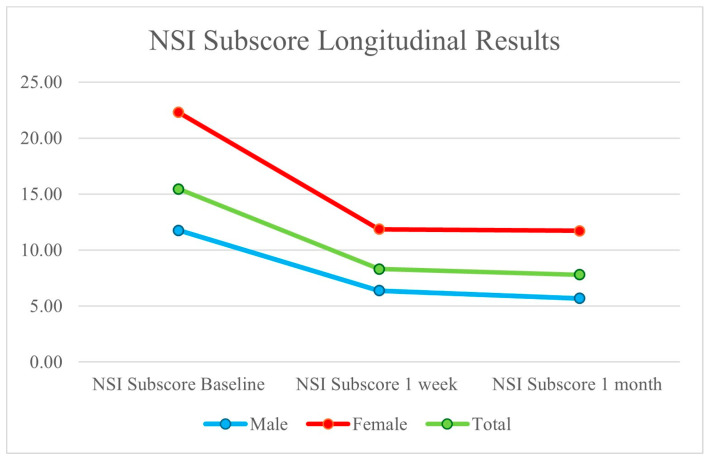
Decrease in patient NSI sub-scores for TBI symptoms following 2LCSB intervention at baseline, one week, and one month. Total scores decreased by nearly 50%, while male patients improved by nearly 4% more than female patients.

The average NSI sub-score was 15.45 (11.77 for males and 22.29 for females), which decreased to 8.30 after one week (6.38 for males and 11.86 for females), and had an average outcome of 7.80 after one month (5.69 for males and 11.71 for females). We saw improvements in both NSI scores and NSI sub-scores at baseline, 1 week, and 1 month; these findings are presented in Figure 1 and Figure 2.

**Figure 3:**
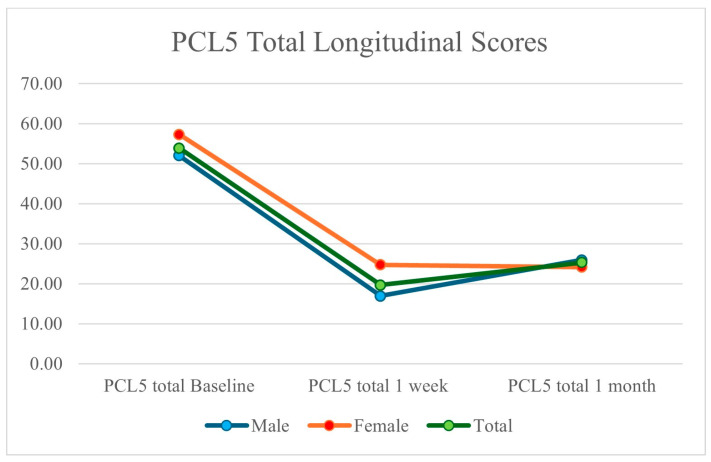
Decrease in patient PCL-5 scores for TBI symptoms following 2LCSB intervention at baseline, one week, and one month. Total scores decreased by over 50%, while female patients improved by nearly 7% more than male patients.

**Figure 4:**
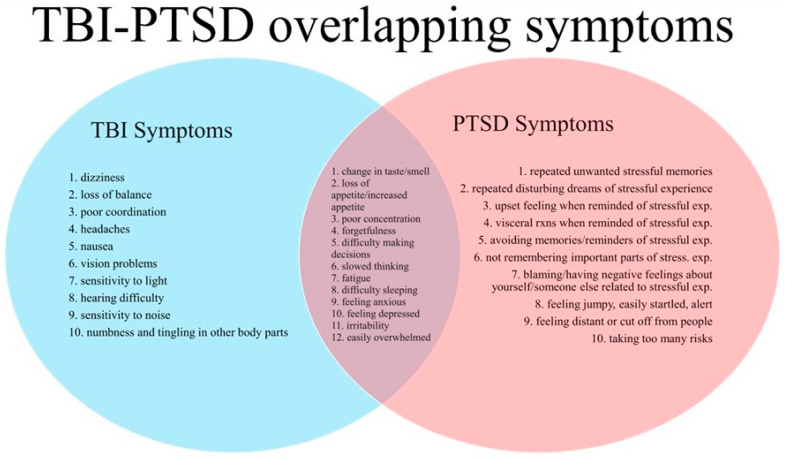
Overlap between TBI and PTSD symptoms as derived from the NSI and PCL-5.

Finally, patients had an average improvement of 53.02% in their PCL-5 scores for PTSD and 54.23% in GAD-7 scores. PCL-5 scores dropped from an average of 53.85 at baseline (52.00 for males and 57.29 for females) to 19.65 at one week (16.92 for males and 24.71 for females) and 25.30 at one month (25.92 for males and 24.14 for females). Average GAD-7 scores decreased from 15.95 at baseline (14.92 for males and 17.86 for females), 5.40 at one week (4.23 for males and 7.57 for females) and 7.30 at one month (6.15 for males and 9.43 for females). The decreases in PCL-5 scores from baseline, 1 week, and 1 month are depicted in Figure 3 and are consistent with previous publications describing improvements in PTSD symptoms following 2LCSB [43,44]. Figure 4 depicts the overlapping (and non-overlapping) symptoms associated with TBI and PTSD as derived from the NSI and PCL-5.

## 4. Discussion

The aim of this study was to determine the efficacy of 2LCSBs for the treatment of TBI-specific symptoms not associated with PTSD. Our 2024 study was the first to identify a relationship between 2LCSB treatment for PTSD in patients with a prior history of TBI. While patients in that study showed improvements, the study failed to address the overlap of TBI and PTSD symptoms and did not provide a dedicated analysis of symptoms present in TBI which were not also present in PTSD [31]. Without specific symptom analysis, the possibility exists that the improvements in total NSI score may have been due largely to improvements in PTSD symptoms.

This work is the first sub-analysis using NSI subset scoring to evaluate symptoms associated with TBI, but not PTSD. Our work in this study found distinct improvements in TBI-specific symptoms using the novel NSI composite sub-score. Both male and female patients saw nearly 50% improvement in their symptoms on the NSI and on the NSI composite score, as seen in Table 1. This indicates that we not only improved patients’ NSI and PCL5-specific symptoms, but there was marked improvements in TBI-specific symptoms following the 2LCSB. This might also suggest that improvements in TBIspecific symptoms and PTSD symptoms as a result of the 2LCSB procedure are well correlated, but further studies of higher rigor are likely needed to make a conclusive determination.

While female patients had higher starting values for the NSI and NSI composite sub-scores, indicating they possibly suffered more severe TBIs, both sexes saw significant improvements in their symptoms. While the incidence of TBI in males is higher than females in civilian populations, more research is required to determine improvements based on the degree of TBI and further determine efficacy of the 2LCSB procedure for these populations [3]. Our results provide positive initial outcomes regarding the use of 2LCSBs for the treatment of TBI symptoms, which may provide relief of persistent TBI-related symptoms as an adjunctive procedure.

This study must be interpreted considering its limitations. The small sample size makes generalizability challenging; the same is true for the retrospective analysis of extant clinical data. The short follow-up period limits the ability to reliably predict longer-term treatment durability, although the relative stability of improvements seen at one week and one month provide a signal that should be evaluated in longer-term studies. Similarly, the lack of formal TBI diagnosis (or characterization of diagnosis as either mild or moderate) limits the utility of these findings. As our conclusions partially rely on an unvalidated subset of the NSI, these findings must be interpreted with caution. Finally, the use of a single keyer for data entry may call those data into question.

## 5. Conclusions

In this limited retrospective case series, the use of bilateral ultrasound-guided 2LCSBs resulted in more than 49.66% improvement in NSI scores at one month post treatment in patients with TBI with a concomitant diagnosis of PTSD. A first-time sub-analysis looking at TBI symptoms which do not overlap with PTSD symptoms showed a 49.51% improvement at one month follow-up. This finding is important since TBI and PTSD are often comorbid and the NSI score alone could potentially fail to detect actual improvement in TBI symptoms. This finding indicates that this treatment may have utility in treating TBI even without comorbid PTSD symptoms. Further studies are needed to assess this treatment modality for the treatment of TBI.

## Figures and Tables

**Table 1:** Average baseline scores, 1-month scores, and percentage improvement from baseline across male, female, and total patients for the NSI, NSI composite sub-score, and PCL-5.

	Male	Female	Total
Avg. NSI at Baseline	41.54	50.00	44.50
Avg. NSI at 1 Month	18.85	29.00	22.40
Avg. NSI % Improvement	54.63%	42.00%	49.66%
Avg. TBI Sub-Score at Baseline	11.77	22.29	15.45
Avg. TBI Sub-Score at 1 Month	5.69	11.71	7.80
Avg. TBI Sub-Score % Improvement	51.63%	47.44%	49.51%
Avg. PCL-5 at Baseline	52.00	57.29	53.85
Avg. PCL-5 at 1 Month	25.92	24.14	25.30
Avg. PCL-5 % Improvement	50.15%	57.86%	53.02%

## Data Availability

The data that support the findings of this study are available from the corresponding author on request due to privacy issues in accordance with the consent provided by the participants. All data are freely accessible.
